# Factors Associated With Changes in Pregnancy Intention Among Women Who Were Mothers of Young Children in New York City Following the COVID-19 Outbreak

**DOI:** 10.1001/jamanetworkopen.2021.24273

**Published:** 2021-09-15

**Authors:** Linda G. Kahn, Leonardo Trasande, Mengling Liu, Shilpi S. Mehta-Lee, Sara G. Brubaker, Melanie H. Jacobson

**Affiliations:** 1 Division of Environmental Pediatrics, Department of Pediatrics, NYU Langone Medical Center, New York, New York; 2 Department of Population Health, NYU Langone Medical Center, New York, New York; 3 Department of Environmental Medicine, NYU Langone Medical Center, New York, New York; 4 NYU Wagner School of Public Service, New York, New York; 5 NYU College of Global Public Health, New York, New York; 6 Department of Obstetrics and Gynecology, NYU Langone Medical Center, New York, New York

## Abstract

**Importance:**

Early evidence shows a decrease in the number of US births during the COVID-19 pandemic, yet few studies have examined individual-level factors associated with pregnancy intention changes, especially among diverse study populations or in areas highly affected by COVID-19 in the US.

**Objective:**

To study changes in pregnancy intention following the outbreak of the COVID-19 pandemic and identify factors possibly associated with these changes.

**Design, Setting, and Participants:**

A cross-sectional, population-based study was conducted among women who were currently pregnant or had delivered a live infant and responded to a survey emailed to 2603 women (n = 1560). Women who were mothers of young children enrolled in the prospective New York University Children’s Health and Environment Study birth cohort were included; women who were not currently pregnant or recently postpartum were excluded.

**Exposures:**

Demographic, COVID-19–related, stress-related, and financial/occupational factors were assessed via a survey administered from April 20 to August 31, 2020.

**Main Outcomes and Measures:**

Pregnancy intentions before the COVID-19 pandemic and change in pregnancy intentions following the outbreak.

**Results:**

Of the 2603 women who were sent the survey, 1560 (59.9%) who were currently pregnant or had delivered a live infant responded, and 1179 women (75.6%) answered the pregnancy intention questions. Mean (SD) age was 32.2 (5.6) years. Following the outbreak, 30 of 61 (49.2%) women who had been actively trying to become pregnant had ceased trying, 71 of 191 (37.2%) women who had been planning to become pregnant were no longer planning, and 42 of 927 (4.5%) women who were neither planning nor trying were newly considering pregnancy. Among those who ceased trying, fewer than half (13 [43.3%]) thought they would resume after the pandemic. Of those pre–COVID-19 planners/triers who stopped considering or attempting pregnancy, a greater proportion had lower educational levels, although the difference was not statistically significant on multivariable analysis (odds ratio [OR], 2.14; 95% CI, 0.92-4.96). The same was true for those with higher stress levels (OR, 1.09; 95% CI, 0.99-1.20) and those with greater financial insecurity (OR, 1.37; 95% CI, 0.97-1.92. Those who stopped considering or attempting pregnancy were more likely to respond to the questionnaire during the peak of the outbreak (OR, 2.04; 95% CI, 1.01-4.11). Of those pre–COVID-19 nonplanners/nontriers who reported newly thinking about becoming pregnant, a smaller proportion responded during the peak, although the finding was not statistically significant on multivariable analysis (OR, 0.52; 95% CI, 0.26-1.03). Likewise, fewer respondents who were financially insecure reported newly considering pregnancy, although the finding was not statistically significant (OR, 0.69; 95% CI, 0.46-1.03). They were significantly less likely to be of Hispanic ethnicity (OR, 0.27; 955 CI, 0.10-0.71) and more likely to have fewer children in the home (OR, 0.62; 95% CI, 0.40-0.98) or self-report a COVID-19 diagnosis (OR, 2.70; 95% CI, 1.31-5.55).

**Conclusions and Relevance:**

In this cross-sectional study of 1179 women who were mothers of young children in New York City, increased stress and financial insecurity owing to the COVID-19 pandemic paralleled a reduction in pregnancy intention in the early months of the pandemic, potentially exacerbating long-term decreases in the fertility rate.

## Key Points

**Question:**

Were there changes in pregnancy intentions among women who were mothers of young children around the peak of the first wave of COVID-19 in New York City?

**Findings:**

In this cross-sectional study of 1179 women in New York City who were mothers of young children, nearly half of those who had been attempting to become pregnant and more than a third who had been thinking about trying before the COVID-19 pandemic stopped in the first few months of the outbreak. Women who responded to a survey during the lockdown were more likely to cease attempts or plans to become pregnant.

**Meaning:**

The results of this study suggest that the outbreak of the COVID-19 pandemic was associated with fewer women planning or attempting to become pregnant; these findings may have long-term effects on fertility rates.

## Teaser

This cross-sectional study examines the intentions on becoming pregnant of women in New York City who were mothers of young children during the first wave of the COVID-19 pandemic.

## Introduction

Accumulating evidence suggests that, in many parts of the world, birth rates have decreased following the COVID-19 outbreak. In June 2020, based on decades of data showing that birth rates track employment and earnings, Kearney and Levine^1^ of the Brookings Institution estimated a deficit of at least 300 000 births in the US in 2021 owing to the economic impact of COVID-19, an estimate they reaffirmed 6 months later after the labor market had started to recover.^2^ Since then, surveys conducted in Europe, in Shanghai, and by the Guttmacher Institute in the US have reported that, in high-income countries, people’s pregnancy intentions have shifted, with 30% to 80% of those who reported an intention to become pregnant within the next year before the COVID-19 outbreak postponing or abandoning their plans.^3,4,5,6^ Recent data suggest that, although the number of births was already decreasing in the US, the decrease between December 2019 and December 2020 was 26% greater than the average year-over-year decrease across the prior 4 months.^7^

COVID-19–related changes in pregnancy intentions have the potential to accelerate the steady decrease in fertility rates seen since the mid-1900s in almost all regions of the world.^8^ On a societal level, reduced fertility rates may have both beneficial and adverse consequences by reducing the strain on the environment while increasing the dependency ratio. On an individual level, people’s decision to delay or abandon childbearing may have negative ramifications across multiple domains. Pregnancy and childbirth become riskier as women age.^9^ Those who delay may experience age-related infertility requiring medical intervention, which is associated with personal^10,11^ and financial^12^ costs, as well as potential health risks to women and children.^13^ In addition, relinquishing the intention to have more children altogether is associated with increased psychological distress^14^ among women.

This study aimed to assess changes in pregnancy intention since the COVID-19 outbreak began among women who were mothers of young children around the peak of the first wave in New York City (NYC), which was then the epicenter of the North American outbreak. We hypothesized that women who had been planning or trying to conceive before the outbreak would report delaying their plans or attempts, and those who experienced the greatest pandemic-related increases in stress and financial strain would be more likely to delay or abandon their pregnancy intentions.

## Methods

### Study Population

The New York University Children’s Health and Environment Study (NYU CHES) is a longitudinal pregnancy cohort that has enrolled women at less than 18 weeks’ gestation since March 2016. Women are recruited at 1 of 3 NYU-affiliated prenatal care sites and followed up through pregnancy, childbirth, and their children’s early years. Questionnaire data and biospecimens are collected in each trimester, at birth, and at periodic intervals throughout infancy and early childhood. NYU CHES is a sociodemographically diverse cohort and, apart from having a higher percentage of women who are Hispanic or married/partnered, reflects the population of pregnant women in NYC.^15^

Beginning April 20, 2020, COVID-19 questionnaires were emailed to NYU CHES participants who were pregnant or had delivered a live infant (n = 2603). Participants could complete the survey in English, Spanish, or Mandarin, either online or on the telephone with a trained study team member fluent in their language. At the time of questionnaire completion, all participants had at least 1 child younger than 3.5 years. Survey administration closed on August 31, 2020. Response rates were calculated according to the American Association for Public Opinion Research reporting guideline for survey studies.^16^ NYU CHES and the COVID-19 questionnaire were approved by the institutional review board of the NYU Grossman School of Medicine. All participants provided written informed consent. Participants did not receive financial compensation. This study followed the Strengthening the Reporting of Observational Studies in Epidemiology (STROBE) reporting guideline.

### Measures

Participants who were not pregnant and had not delivered since March 1, 2020, were asked a series of questions about their pre–COVID-19 and current pregnancy intentions (Box). Potential factors associated with pregnancy intention included age, self-reported race and ethnicity based on US Census categories (a proxy for unmeasured stressors related to social position), insurance status, annual household income, educational level, and the number of children aged 18 years or younger living in the home. Participants were asked to rate their pre–COVID-19 and current financial security,^17^ as well as their stress level during the 2 weeks preceding the questionnaire using the validated 4-item Perceived Stress Scale (score range, 0-16, with higher scores denoting greater stress).^18^ They were also asked about COVID-19 symptoms, diagnoses, and testing histories among household members since March 1, 2020 (the day of the first confirmed COVID-19 diagnosis in NYC), whether they or another adult in the household had lost their job due to the pandemic, and whether any adult in the household was an essential worker. The date of survey completion was dichotomized at June 1, 2020, when NYC began reopening after lockdown.

Box. **COVID-19–Related Pregnancy Intention Questions, New York University Children’s Health and Environment Study, 2020**

1. [All]: **Before the COVID-19 epidemic**, were you thinking about becoming pregnant in the next 6 to 12 months?
  - If yes, **Before the COVID-19 epidemic**, were you actively trying to become pregnant?
    - If yes, Are you still trying to become pregnant?
  - If no, Do you plan to resume trying to become pregnant once the epidemic is over?
2. [All]: Are you **currently** thinking about becoming pregnant in the next 6 to 12 months?
  - If yes, Are you actively trying to become pregnant right now?

### Statistical Analysis

Three groups were derived from the series of questions about pregnancy intentions. Pre–COVID-19 planners were the first group; before the pandemic, these women were thinking about becoming pregnant within the next 6 to 12 months. The second group comprised pre–COVID-19 triers: women who, before the pandemic, were actively trying to become pregnant. The third group comprised pre–COVID-19 nonplanners/nontriers; before the pandemic, these women were not thinking about becoming pregnant within the next 6 to 12 months.

Outcomes were defined as changes in the intentions of these women. The outcome groups included, among pre–COVID-19 planners, women who were no longer thinking about becoming pregnant within the next 6 to 12 months at the time of questionnaire completion; among pre–COVID-19 triers, women who were no longer trying to become pregnant at the time of questionnaire completion; and, among pre–COVID-19 nonplanners/nontriers, women who were newly thinking about or trying to become pregnant at the time of questionnaire completion. In each case, the comparison group consisted of women who did not change their intentions.

Participant demographic, COVID-19–related, stress-related, and financial/occupational characteristics were compared across strata of pregnancy intention outcomes. Differences in categorical variables were analyzed with χ^2^ tests, and *t* tests were used to assess differences in continuous variables. Simple logistic regression models were fit to estimate crude associations and multivariable logistic models were fit to estimate mutually adjusted associations between participant characteristics and pregnancy intention outcomes. Variables were selected according to results of bivariate analyses. Final models included covariates that, with 2-tailed testing, were significantly associated (*P* < .05) with the outcome (changing pregnancy intention) among at least 1 of the 3 groups and not associated with other variables. First, a model was fit among pre–COVID-19 planners and triers. These outcomes were collapsed owing to small sample size of those actively trying to become pregnant. Second, a model was fit among pre–COVID-19 nonplanners/nontriers. Sensitivity analyses were considered by fitting models among planners and triers separately and excluding women outside the NYC metropolitan area.

## Results

Of the 2603 women contacted, 1560 (59.9%) completed the COVID-19 questionnaire by August 31, 2020. After excluding those who were pregnant (n = 228), had recently given birth (n = 103), and were missing responses to these 2 questions (n = 40) and pregnancy intention questions (n = 10), 1179 women (75.6% of respondents) remained in the analytic sample. Mean (SD) age was 32.2 (5.6) years. Respondents were sociodemographically similar to the underlying cohort (eTable 1 in the Supplement).

A total of 191 women (16.2%) reported that before the outbreak they were thinking about becoming pregnant within the next 6 to 12 months but not yet trying, 61 (5.2%) reported actively trying, and 927 (78.6%) reported not thinking about becoming pregnant within the next 6 to 12 months (eTable 2 in the Supplement). Women in the 3 groups were comparable in age and pre–COVID-19 stress levels but differed according to a number of other baseline characteristics. Women thinking about becoming pregnant within the next 6 to 12 months were most likely to be White (108 [56.5%]), whereas Hispanic women represented most of those not considering pregnancy (544 [58.7%]). Those who were actively trying to become pregnant (32 [52.5%]) or thinking about becoming pregnant (147 [77.0%]) were more likely to have private health insurance; most of those not considering pregnancy had public health insurance (552 [59.6%]). Women contemplating pregnancy were more likely to have annual household incomes greater than or equal to $100 000 (127 [66.5%]), at least a bachelor’s degree (151 [79.1%]), and the highest level of financial security (116 [60.7%]) compared with other groups. Women who were not considering pregnancy had more children living in the home (mean [SD], 2.0 [1.1]) than those who were thinking about becoming pregnant (mean [SD], 1.3 [0.8]) or trying to become pregnant (mean [SD], 1.6 [0.8]).

When asked about their current pregnancy intentions at the time of questionnaire completion (ie, 6 weeks to 6 months following the COVID-19 outbreak in NYC), 71 of 191 women (37.2%) who had been thinking about becoming pregnant no longer were, 30 of 61 (49.2%) women who had been actively trying had ceased, and 42 of 927 (4.5%) women who had not been considering pregnancy were now considering it (Figure). Among 120 women still contemplating pregnancy, 20 (16.7%) had started actively trying. Among 30 women who had been trying and stopped, 13 (43.3%) thought they would resume trying, 4 (13.3%) thought they would not resume trying, and 13 (43.3%) were uncertain. Among 42 women newly thinking about becoming pregnant, only 1 (2.4%) was actively trying.

**Figure.**
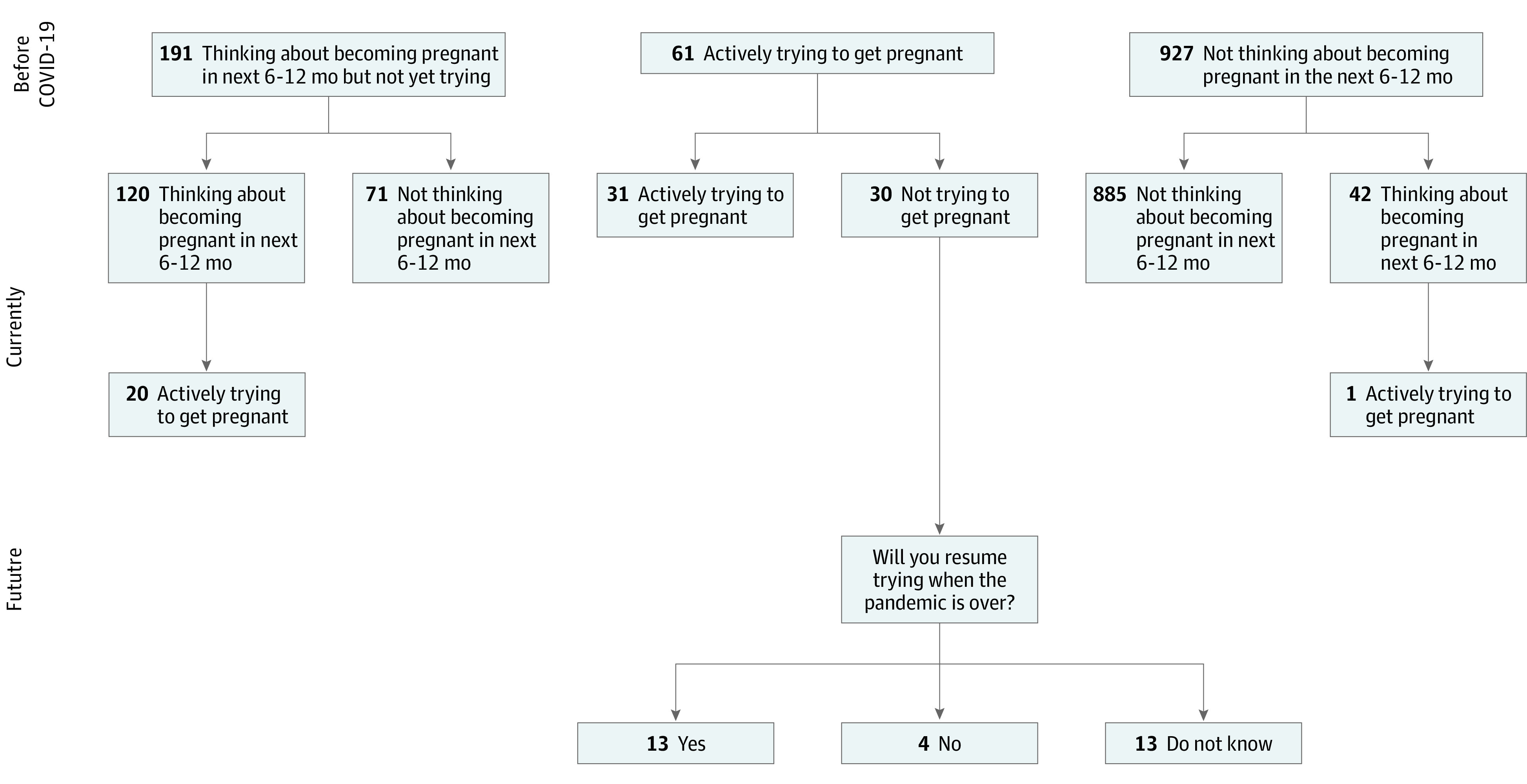
Trajectory of Pregnancy Intentions Spanning the Early COVID-19 Outbreak in New York City Among 1179 New York University Children’s Health and Environment Study Participants, 2020

In bivariate analyses focusing on women who changed their minds after the pandemic outbreak, among pre–COVID-19 planners, a greater proportion of women with public insurance (22 [31.4%]), lower income (25 [35.2%]), lower educational level (25 [35.2%]), lower current financial security (10 [14.1%]), and COVID-19–related job loss in the household (41 [57.7%]) stopped contemplating pregnancy within the next 6 to 12 months (Table 1). Those no longer contemplating pregnancy were also more likely to be younger (mean [SD], 32.3 [5.8] years), have more children at home, report higher perceived stress, and have taken the survey before June 1, 2020. Among those actively trying to become pregnant before the COVID-19 pandemic, Hispanic women (19 [63.3%]), women with lower educational levels (13 [43.3%]), and women with more children living in the home (mean [SD], 1.8 [0.9]) were more likely to have stopped. Among pre–COVID-19 nonplanners/nontriers, women who reported newly thinking about becoming pregnant were more likely to be non-Hispanic White (18 [42.9%]); have private insurance (23 [54.8%]), high income (22 [52.4%]), high educational level (24 [58.5%]), and current financial security (22 [53.7%]); have fewer children in the home (mean [SD], 1.6 [0.8]); and have a self-reported diagnosis of COVID-19. Women in this group were also less likely to have completed the survey before June 1, 2020.

**Table 1.** Characteristics of Study Population by COVID-19–Related Fertility Intention Changes

Characteristic	Total, No. (%) (n = 1179)	Thinking about becoming pregnant before COVID-19, No. (%) (n = 191)		*P* value	Actively trying to become pregnant before COVID-19, No. (%) (n = 61)		*P* value	Not thinking about becoming pregnant before COVID-19, No. (%) (n = 927)		*P* value
		Still thinking (n = 120 [63%])	No longer thinking (n = 71 [37%])		Still trying (n = 31 [51%])	No longer trying (n = 30 [49%])		Still not thinking (n = 885 [95%])	Thinking (n = 42 [5%])	
Demographics										
Age, mean (SD), y	32.2 (5.6)	33.1 (4.0)	31.2 (5.0)	.01	33.0 (4.5)	31.0 (6.5)	.17	32.3 (5.8)	31.0 (5.4)	.15
Race and ethnicity										
Hispanic	622 (52.9)	25 (21.0)	24 (33.8)	.14	10 (32.3)	19 (63.3)	.04	530 (59.9)	14 (33.3)	.003
Non-Hispanic										
White	372 (31.6)	71 (59.7)	37 (52.1)		12 (38.7)	8 (26.7)		226 (25.5)	18 (42.9)	
Black, Asian, and other	183 (15.6)	23 (19.3)	10 (14.1)		9 (29.0)	3 (10.0)		128 (14.5)	10 (23.8)	
Insurance status										
Public	623 (53.3)	21 (17.5)	22 (31.4)	.03	12 (38.7)	16 (55.2)	.20	533 (60.7)	19 (45.2)	.05
Private	547 (46.8)	99 (82.5)	48 (68.6)		19 (61.3)	13 (44.8)		345 (39.3)	23 (54.8)	
Annual household income, $										
<100 000	415 (35.2)	21 (17.5)	25 (35.2)	.002	10 (32.3)	13 (43.3)	.19	335 (37.9)	11 (26.2)	.01
≥100 000	427 (36.2)	90 (75.0)	37 (52.1)		15 (48.4)	9 (30.0)		254 (28.7)	22 (52.4)	
Do not know	337 (28.6)	9 (7.5)	9 (12.7)		6 (19.4)	8 (26.7)		296 (33.4)	9 (21.4)	
Educational level										
<Bachelor's degree	570 (49.4)	12 (10.3)	25 (35.2)	<.001	9 (29.0)	13 (43.3)	.03	490 (56.7)	17 (41.5)	.05
≥Bachelor's degree	584 (50.6)	105 (89.7)	46 (64.8)		22 (71.0)	17 (56.7)		374 (43.3)	24 (58.5)	
Children (≤18 y) living in home, mean (SD)	1.9 (1.0)	1.2 (0.8)	1.5 (0.8)	.04	1.4 (0.6)	1.8 (0.9)	.05	2.1 (1.1)	1.6 (0.8)	<.001
COVID-19 related										
Diagnosis (self)	221 (18.7)	21 (17.5)	17 (23.9)	.28	4 (12.9)	6 (20.0)	.45	158 (17.9)	15 (35.7)	.004
Partner diagnosis	146 (13.8)	20 (17.4)	4 (6.0)	.03	6 (20.0)	4 (17.4)	.81	103 (13.1)	9 (23.1)	.08
Child diagnosis	126 (10.8)	15 (12.9)	7 (10.1)	.57	4 (12.9)	4 (13.3)	.96	92 (10.5)	4 (9.5)	.84
COVID-19 hospitalization in household	11 (0.9)	0	1 (1.4)	.19	0	1 (3.3)	.31	9 (1.0)	0	.51
Stress related										
PSS-4 score, mean (SD)^a^	6.3 (2.8)	6.0 (2.8)	7.3 (3.1)	.01	6.2 (2.7)	6.8 (3.1)	.41	6.3 (2.8)	6.0 (2.7)	.62
Pre–COVID-19 financial security										
Comfortable with extra	436 (37.2)	77 (64.2)	39 (54.9)	.13	14 (45.2)	10 (34.5)	.69	274 (31.2)	22 (53.7)	.01
Enough but no extra	522 (44.6)	36 (30.0)	22 (31.0)		13 (41.9)	14 (48.3)		421 (47.9)	16 (39.0)	
Have to cut back or cannot make ends meet	213 (18.2)	7 (5.8)	10 (14.1)		4 (12.9)	5 (17.2)		184 (20.9)	3 (7.3)	
Current financial security										
Comfortable with extra	269 (22.9)	63 (52.5)	21 (29.6)	.002	9 (29.0)	4 (13.8)	.36	158 (17.9)	14 (34.2)	.01
Enough but no extra	312 (26.6)	29 (24.2)	13 (18.3)		8 (25.8)	9 (31.0)		239 (27.1)	14 (34.2)	
Have to cut back or cannot make ends meet	592 (50.5)	28 (23.3)	37 (52.1)		14 (45.2)	16 (55.2)		484 (54.9)	13 (31.7)	
Job loss (self or other adult in home)	764 (64.8)	51 (42.5)	41 (57.7)	.04	19 (61.3)	19 (63.3)	.87	610 (68.9)	24 (57.1)	.11
Essential worker in household	289 (24.5)	33 (27.5)	23 (32.4)	.47	7 (22.6)	9 (30.0)	.51	208 (23.5)	9 (21.4)	.76
Took questionnaire before June 1, 2020	926 (78.5)	85 (70.8)	60 (84.5)	.03	24 (77.4)	23 (76.7)	.94	707 (79.9)	27 (64.3)	.02

Abbreviation: PSS, Perceived Stress Scale.

^a^ PSS-4 scored from 0 to 16, with higher scores denoting greater stress.

Among pre–COVID-19 planners and triers (n = 252, combined for the purpose of regression analysis), 40.1% (n = 101) ceased trying to become or considering becoming pregnant. These women tended to have lower educational levels (odds ratio [OR] for <college vs ≥college, 2.14; 95% CI, 0.92-4.96); higher stress (OR per 1-unit increase in PSS-4 score, 1.09; 95% CI, 0.99, 1.20), and higher financial insecurity (OR per 1-unit increase in financial insecurity scale, 1.37; 95% CI, 0.97, 1.92), although these findings were not statistically significant owing to correlation among covariates (Table 2). When we considered pre–COVID-19 planners and triers separately, the results were similar (eTable 3 and eTable 4 in the Supplement). Of those pre–COVID-19 nonplanners/nontriers who reported newly thinking about becoming pregnant at the time of the questionnaire, a smaller proportion responded during the peak (OR, 0.52; 95% CI, 0.26-1.03). Likewise fewer respondents who were financially insecure reported newly considering pregnancy, although the finding was not statistically significant (OR, 0.69; 95% CI, 0.46-1.03); however, they were significantly less likely to have Hispanic ethnicity (OR, 0.27; 95% CI, 0.10-0.71) and more likely to have fewer children in the home (OR, 0.62; 95% CI, 0.40-0.98); and were more likely to self-report a COVID-19 diagnosis (OR, 2.70; 95% CI, 1.31-5.55) (Table 3). The results of sensitivity analyses excluding women who lived outside the NYC metropolitan area were similar to our main findings (eTable 5 and eTable 6 in the Supplement).

**Table 2.** Odds Ratios From Logistic Regression Models Regarding Changing Fertility Intention Among 252 Participants

Variable	No longer trying or thinking vs still trying or thinking, OR (95% CI)	
	Crude	Adjusted
Maternal age	0.92 (0.87-0.97)	0.95 (0.89-1.01)
Hispanic (vs not Hispanic)	2.44 (1.41-4.21)	1.00 (0.48-2.05)
<College degree (vs ≥college)	4.31 (2.34-7.91)	2.14 (0.92-4.96)
No. of children in the home	1.70 (1.19-2.45)	1.23 (0.84-1.82)
COVID-19 diagnosis (self) (vs not)	1.49 (0.79-2.80)	1.37 (0.67-2.77)
PSS-4 score	1.14 (1.04-1.25)	1.09 (0.99-1.20)
Current financial insecurity	1.81 (1.39-2.34)	1.37 (0.97-1.92)
Took questionnaire before June 1, 2020	1.78 (0.95-3.31)	2.04 (1.01-4.11)

Abbreviations: OR, odds ratio; PSS, Perceived Stress Scale.

**Table 3.** Odds Ratios From Logistic Regression Models for Factors of Thinking of Becoming Pregnant Among Those Who Were Not Thinking About It Before COVID-19 Among 927 Participants

Variable	Thinking of becoming pregnant vs still not, OR (95% CI)	
	Crude	Adjusted
Maternal age	0.96 (0.91-1.01)	0.95 (0.89-1.01)
Hispanic (vs not Hispanic)	0.33 (0.17-0.64)	0.27 (0.10-0.71)
<College degree (vs ≥college)	0.54 (0.29-1.02)	1.79 (0.67-4.75)
No. of children in the home	0.54 (0.36-0.81)	0.62 (0.40-0.98)
COVID-19 diagnosis (self) (vs not)	2.56 (1.33-4.92)	2.70 (1.31-5.55)
PSS-4 score	0.97 (0.87-1.09)	0.97 (0.86-1.09)
Current financial insecurity	0.56 (0.40-0.78)	0.69 (0.46-1.03)
Completed questionnaire before June 1, 2020	0.45 (0.24-0.87)	0.52 (0.26-1.03)

Abbreviations: OR, odds ratio; PSS, Perceived Stress Scale.

## Discussion

The COVID-19 pandemic was associated with altered pregnancy intentions among women who were mothers of young children in this large, diverse NYC cohort. Nearly half of women who had been trying to become pregnant and more than a third who had been thinking about trying stopped in the first few months of the COVID-19 outbreak, and only a small proportion newly contemplated becoming pregnant. Among those who ceased trying, fewer than half were sure they would resume trying once the pandemic was over, suggesting many may be abandoning rather than delaying plans to expand their families, with potential long-term consequences for the overall fertility rate.

Our findings align with those of a national internet-based survey of 2009 sexually active cisgendered women aged 18 to 49 years conducted by the Guttmacher Institute from April 30 to May 6, 2020. Lindberg et al^6^ reported that more than 40% of respondents had changed their plans about when and how many children to have, including 34% who wanted to become pregnant later or have fewer children because of the pandemic. Lower-income and non-Hispanic Black and Hispanic women were more likely to delay pregnancy or reduce their desired number of children. Although this study was larger than ours and included nulliparous as well as parous women across the reproductive life span, it was based on a convenience sample and was unable to explore in comparable depth associations with household-level or COVID-19–related factors.

In our survey we found that women who ceased to contemplate or attempt pregnancy following the outbreak tended to be less financially secure and that the opposite was true for women who were newly considering pregnancy, although results were not statistically significant, possibly because of the small sample size. This finding is similar to what was seen during the 2008 recession, when data from the US National Vital Statistics System showed that the total fertility rate precipitously decreased following 2 decades of hovering around replacement level.^19^ The fertility rate has since continued to decrease, reflecting changes in fertility preferences, and reached an historic low of 1.64 births per woman of reproductive age in 2020—the most recent data available. Even as the gap between 2019 and 2020 monthly births had been widening throughout 2020, December, which was the earliest it would be reasonable to expect to see an association between COVID-19 and pregnancy intention (9 months following the first widespread outbreaks in the US), stood out, with a 7.7% decrease compared with 2019, 2 percentage points higher than the average decrease across the previous 4 months.^7^ Reports from both the Brookings Institution^20^ and the Center for American Progress^21^ highlight the many ways in which the economic effects resulting from the pandemic have disproportionately affected women, especially working mothers. A survey by The Pew Charitable Trusts found that, between February and August 2020, the time frame of our study, 3 times as many women who were mothers of small children lost their jobs as men who were fathers^22^; in September, the discrepancy increased to 4 times as many.^23^ This level contrasts with the 2008 recession, in which more men lost jobs. Although more women who were Black lost jobs than those who were White in both the 2008 and COVID-19 downturns, the current race gap is greater, underscoring the intersectionality of race and sex when it comes to economic disadvantage.^23^

Women who were no longer considering or trying to become pregnant tended to report higher stress levels, although findings were not statistically significant, possibly because of the small sample size. The 4-item Perceived Stress Scale asks about feelings of control, confidence, optimism, and being overwhelmed, all of which are relevant to the pressures of raising young children under pandemic restrictions, including remote schooling and insufficient childcare. Other potential sources of stress include anxiety, depression, and domestic violence, which have been shown to have increased among pregnant women and mothers since the outbreak.^24^ In addition, respondents’ feelings may have been affected by heightened tension around racial issues prevalent throughout the spring and summer of 2020 and the intensity of Black Lives Matter protests following the death of George Floyd. Although our survey did not specifically interrogate perceived racial discrimination and anxiety around racism, the temporal coincidence of increased stress among those no longer considering pregnancy with the concurrent national focus on racial injustice suggests that this may be an avenue for future research.

Women least likely to cease contemplating or attempting pregnancy and those most likely to newly consider becoming pregnant in our study tended to be high-income, highly educated, and non-Hispanic White individuals. This finding parallels other evidence suggesting that those with financial security have continued to actively pursue pregnancy despite the pandemic, most obviously in the area of assisted reproduction,^25,26^ where use rates align with income and race, especially in states where insurance coverage is not mandated.^27,28^

### Strengths and Limitations

The greatest strength of our study is its inclusion of data from a large, diverse group of women who were mothers of young children during and immediately following the first wave of the COVID-19 outbreak in NYC. Although our results are not generalizable to nulliparous women, they are generalizable to the population of women who had recently given birth in NYC and may be applicable to comparable populations during COVID-19 epidemic peaks.

The study also has limitations, the main one being the lack of power owing to the low prevalence of women reporting planning to become pregnant or actively trying before the pandemic. Although this study was nested within a longitudinal cohort, we did not have a pre–COVID-19 assessment of pregnancy intention in our database. These responses may be affected by recall bias, as women were asked to recollect their pregnancy intentions up to 6 months after the outbreak and under different circumstances. We hypothesize that any recall bias would most likely lead to underreporting of pre–COVID-19 planning or trying, which would result in a reduced sample size for analyses of pre–COVID-19 planners and triers. Although we did not explicitly ask whether changes in pregnancy intention were due to the COVID-19 outbreak, the phrasing, bolding, and sequence of the survey questions suggest that it is reasonable to infer that these changes may be in large part attributable to the pandemic. Pregnancy intention questions were not asked of pregnant women or those who had given birth since the outbreak, because we assumed few, if any, women would be planning another pregnancy so soon. If they had, we have no reason to believe the change in their intentions would have differed from those of other participants. Non-Hispanic Black women were underrepresented in our study relative to their population in NYC owing to the demographics of the hospitals where NYU CHES recruits, preventing us from drawing more nuanced inferences on race. Because we designed our study before the increased urgency of Black Lives Matter activism, we did not consider its potential relevance to our study and did not ask questions that might have illuminated the possible association between stress due to racial tension and discrimination and pregnancy intention.

## Conclusions

In our large, diverse NYC cohort of women who were mothers of young children, there were fewer women planning or attempting to become pregnant again following the outbreak of COVID-19 and there was substantial uncertainty around whether their intentions would change after the crisis passed. The stress and increased financial insecurity among mothers of young children in the early months of the pandemic could exacerbate declines in the fertility rate over the long term. Future studies might explore whether pregnancy intentions have rebounded as conditions improved and whether patterns have differed across racial and ethnic groups.

## 

## Notes

Supplement. **eTable 1.** Demographic Profiles of New York University Children's Health and Environment Study (CHES) Participants vs CHES COVID-19 Survey Respondents **eTable 2.** Characteristics of Study Population by Pre-COVID-19 Fertility Intentions **eTable 3.** Crude and Adjusted Odds Ratios (OR) and 95% Confidence Intervals (CI) From Logistic Regression Models for Predictors of Changing Fertility Intention From Thinking About Trying to Get Pregnant Before COVID-19 to No Longer Thinking About Trying to Get Pregnant After COVID-19 (n=191) **eTable 4.** Crude and Adjusted Odds Ratios (OR) and 95% Confidence Intervals (CI) From Logistic Regression Models for Predictors of Changing Fertility Intention From Trying to Get Pregnant Before COVID-19 to No Longer Trying to Get Pregnant After COVID-19 (n=61) **eTable 5.** Adjusted Odds Ratios (OR) and 95% Confidence Intervals (CI) From Logistic Regression Models for Predictors of Changing Fertility Intention From Trying or Thinking About Trying to Get Pregnant Before COVID-19 to No Longer Trying or Thinking About Trying to Get Pregnant After COVID-19 Excluding Those Living Outside the New York City Metro Area (n=214) **eTable 6.** Adjusted Odds Ratios (OR) and 95% Confidence Intervals (CI) From Logistic Regression Models for Predictors of Thinking of Becoming Pregnant Among Those Who Were Not Thinking About It Before COVID-19 Excluding Those Living Outside the New York City Metro Area (n=817)
