# Supplementary material for: Factors Associated With Changes in Pregnancy Intention Among Women Who Were Mothers of Young Children in New York City Following the COVID-19 Outbreak
Source: JAMA Netw Open. 2021 Sep 15;4(9):e2124273. doi: 10.1001/jamanetworkopen.2021.24273 (PMC8444024; doi:10.1001/jamanetworkopen.2021.24273)
Supplement: Supplement. — eTable 1. Demographic Profiles of New York University Children's Health and Environment Study (CHES) Participants vs CHES COVID-19 Survey Respondents eTable 2. Characteristics of Study Population by Pre-COVID-19 Fertility Intentions eTable 3. Crude and Adjusted Odds Ratios (OR) and 95% Confidence Intervals (CI) From Logistic Regression Models for Predictors of Changing Fertility Intention From Thinking About Trying to Get Pregnant Before COVID-19 to No Longer Thinking About Trying to Get Pregnant After COVID-19 (n=191) eTable 4. Crude and Adjusted Odds Ratios (OR) and 95% Confidence Intervals (CI) From Logistic Regression Models for Predictors of Changing Fertility Intention From Trying to Get Pregnant Before COVID-19 to No Longer Trying to Get Pregnant After COVID-19 (n=61) eTable 5. Adjusted Odds Ratios (OR) and 95% Confidence Intervals (CI) From Logistic Regression Models for Predictors of Changing Fertility Intention From Trying or Thinking About Trying to Get Pregnant Before COVID-19 to No Longer Trying or Thinking About Trying to Get Pregnant After COVID-19 Excluding Those Living Outside the New York City Metro Area (n=214) eTable 6. Adjusted Odds Ratios (OR) and 95% Confidence Intervals (CI) From Logistic Regression Models for Predictors of Thinking of Becoming Pregnant Among Those Who Were Not Thinking About It Before COVID-19 Excluding Those Living Outside the New York City Metro Area (n=817) [file jamanetwopen-e2124273-s001.pdf]

Supplementary Online Content

Kahn LG, Trasande L, Liu M, Mehta-Lee SS, Brubaker SG, Jacobson MH. Factors associated with changes in pregnancy intention among women who were mothers of young children in New York City following the COVID-19 outbreak. *JAMA Netw Open*. 2021;4(9):e2124273. doi:10.1001/jamanetworkopen.2021.24273

eTable 1. Demographic Profiles of New York University Children's Health and Environment Study (CHES) Participants vs. CHES COVID-19 Survey Respondents

eTable 2. Characteristics of Study Population by Pre-COVID-19 Fertility Intentions

eTable 3. Crude and Adjusted Odds Ratios (OR) and 95% Confidence Intervals (CI) From Logistic Regression Models for Predictors of Changing Fertility Intention From Thinking About Trying to Get Pregnant Before COVID-19 to No Longer Thinking About Trying to Get Pregnant After COVID-19 (n=191)

eTable 4. Crude and Adjusted Odds Ratios (OR) and 95% Confidence Intervals (CI) From Logistic Regression Models for Predictors of Changing Fertility Intention From Trying to Get Pregnant Before COVID-19 to No Longer Trying to Get Pregnant After COVID-19 (n=61)

eTable 5. Adjusted Odds Ratios (OR) and 95% Confidence Intervals (CI) From Logistic Regression Models for Predictors of Changing Fertility Intention From Trying or Thinking About Trying to Get Pregnant Before COVID-19 to No Longer Trying or Thinking About Trying to Get Pregnant After COVID-19 Excluding Those Living Outside the New York City Metro Area (n=214)

eTable 6. Adjusted Odds Ratios (OR) and 95% Confidence Intervals (CI) From Logistic Regression Models for Predictors of Thinking of Becoming Pregnant Among Those Who Were Not Thinking About It Before COVID-19 Excluding Those Living Outside the New York City Metro Area (n=817)

This supplementary material has been provided by the authors to give readers additional information about their work.

© 2021 Kahn LG et al. *JAMA Network Open.*

eTable 1. Demographic profiles of New York University Children's Health and Environment Study (CHES) participants vs. CHES COVID-19 survey respondents

	Total CHES COVID-19 survey respondents (n=1560)	Total CHES cohort (n=2193)
Age, mean (SD)	32.1 (5.6)	31.8 (5.6)
Race/ethnicity, n (%)		
Hispanic	781 (50.2)	1071 (49.0)
White	518 (33.3)	727 (33.3)
Black	70 (4.5)	130 (6.0)
Asian	141 (9.1)	193 (8.8)
Other/Multiple	46 (3.0)	64 (2.9)
Insurance status, n (%)		
Public	770 (50.3)	1139 (52.5)
Private	761 (49.7)	1032 (47.5)
Annual household income, n (%)		
<$30,000	272 (17.4)	362 (16.5)
$30,000 to $100,000	270 (17.3)	425 (19.4)
≥$100,000	601 (38.5)	805 (36.7)
Don't know	417 (26.7)	601 (27.4)
Education, n (%)		
High school or less	504 (33.1)	704 (32.5)
Some college	223 (14.6)	362 (16.7)
Bachelor's degree	365 (24.0)	498 (23.0)
Postgraduate	432 (28.4)	602 (27.8)

© 2021 Kahn LG et al. *JAMA Network Open.*

**eTable 2**. Characteristics of study population by pre-COVID-19 fertility intentions								
Before COVID	Thinking about becoming pregnant (n=191)		Actively trying to become pregnant (n=61)		Not thinking about becoming pregnant (n=927)		p	
Age, years mean (SD)	32.4	4.5	32.0	5.6	32.2	5.8	0.89	
Race/ethnicity, n (%)							<0.01	
Hispanic	49	25.7	29	47.5	544	58.7		
Non-Hispanic White	108	56.5	20	32.8	244	26.3		
Non-Hispanic Black	10	5.2	3	4.9	43	4.6		
Non-Hispanic Asian	20	10.5	7	11.5	67	7.2		
Non-Hispanic Other/Multiple	3	1.6	2	3.3	28	3.0		
Insurance status, n (%)							<0.01	
Public	43	22.5	28	45.9	552	59.6		
Private	147	77.0	32	52.5	368	39.7		
Annual household income, n (%)							<0.01	
<$30,000	16	8.4	8	13.1	183	19.7		
$30,000 to $100,000	30	15.7	15	24.6	163	17.6		
≥$100,000	127	66.5	24	39.3	276	29.8		
Don't know	18	9.4	14	23.0	305	32.9		
Education, n (%)							<0.01	
High school or less	16	8.4	18	29.5	359	38.7		
Some college	21	11.0	8	13.1	148	16.0		
Bachelor's degree	64	33.5	21	34.4	181	19.5		
Postgraduate	87	45.6	14	23.0	217	23.4		
Children (≤18 years) living in house, mean (SD)	1.3	0.8	1.6	0.8	2.0	1.1	<0.01	
Pre-COVID financial security, n (%)							<0.01	
Comfortable with extra	116	60.7	24	39.3	296	31.9		
Enough but no extra	58	30.4	27	44.3	437	47.1		
Have to cut back	10	5.2	5	8.2	123	13.3		
Cannot make ends meet	7	3.7	4	6.6	64	6.9		
Took questionnaire before June 1, 2020	145	75.9	47	77.1	734	79.2	0.58	

© 2021 Kahn LG et al. *JAMA Network Open.*

eTable 3. Crude and adjusted odds ratios (OR) and 95% Confidence Intervals (CI) from logistic regression models for predictors of changing fertility intention from thinking about trying to get pregnant before COVID-19 to no longer thinking about trying to get pregnant after COVID-19 (n=191).

	No longer thinking vs. still thinking			
	**Crude OR (95% CI)**	**p**	**Adjusted OR (95% CI)**	**p**
Maternal age	0.91 (0.85, 0.97)	0.01	0.93 (0.85, 1.01)	0.09
Hispanic (vs. not Hispanic)	1.92 (0.99, 3.72)	0.05	0.72 (0.30, 1.74)	0.47
< College degree (vs. ≥ college)	4.75 (2.20, 10.27)	<0.01	2.20 (0.77, 6.28)	0.14
Number of children in the home	1.52 (1.00, 2.30)	0.05	1.16 (0.75, 1.80)	0.51
COVID diagnosis (self) (vs. not)	1.48 (0.72, 3.05)	0.28	1.30 (0.57, 2.96)	0.53
PSS-4 score	1.16 (1.05, 1.29)	0.01	1.13 (1.00, 1.27)	0.05
Current financial security (less vs. more)	1.88 (1.39, 2.55)	<0.01	1.47 (0.98, 2.21)	0.06
Took questionnaire before June 1, 2020	2.05 (1.06, 4.77)	0.04	2.14 (0.92, 4.94)	0.08

© 2021 Kahn LG et al. *JAMA Network Open.*

eTable 4. Crude and adjusted odds ratios (OR) and 95% Confidence Intervals (CI) from logistic regression models for predictors of changing fertility intention from trying to get pregnant before COVID-19 to no longer trying to get pregnant after COVID-19 (n=61)

	No longer trying vs. still trying			
	**Crude OR (95% CI)**	**p**	**Adjusted OR (95% CI)**	**p**
Maternal age	0.94 (0.85, 1.03)	0.17	0.98 (0.87, 1.10)	0.71
Hispanic (vs. not Hispanic)	3.63 (1.26, 10.44)	0.02	2.68 (0.60, 12.0)	0.20
< College degree (vs. ≥ college)	3.20 (1.11, 9.22)	0.03	1.34 (0.27, 6.71)	0.72
Number of children in the home	2.00 (1.00, 4.01)	0.05	1.70 (0.74, 3.89)	0.21
COVID diagnosis (self) (vs. not)	1.69 (0.43, 6.70)	0.46	1.90 (0.39, 9.16)	0.42
PSS-4 score	1.08 (0.90, 1.29)	0.41	1.05 (0.85, 1.29)	0.68
Current financial security (less vs. more)	1.49 (0.89, 2.52)	0.13	0.91 (0.44, 1.89)	0.79
Took questionnaire before June 1, 2020	0.96 (0.29, 3.16)	0.94	1.99 (0.44, 9.04)	0.37

© 2021 Kahn LG et al. *JAMA Network Open.*

**eTable 5.** Adjusted odds ratios (OR) and 95% Confidence Intervals (CI) from logistic regression models for predictors of changing fertility intention from trying or thinking about trying to get pregnant before COVID-19 to no longer trying or thinking about trying to get pregnant after COVID-19 excluding those living outside the New York City metro area (n=214)		
	No longer trying or thinking vs. still trying or thinking	
	**Adjusted OR (95% CI)**	**p**
Maternal age	0.96 (0.89, 1.03)	0.22
Hispanic (vs. not Hispanic)	0.99 (0.46, 2.16)	0.99
< College degree (vs. ≥ college)	2.33 (0.93, 5.84)	0.07
Number of children in the home	1.78 (1.11, 2.85)	0.02
COVID diagnosis (self) (vs. not)	1.26 (0.59, 2.69)	0.55
PSS-4 score	1.05 (0.94, 1.17)	0.39
Current financial insecurity	1.23 (0.85, 1.78)	0.28
Took questionnaire before June 1, 2020	1.72 (0.79, 3.76)	0.18

© 2021 Kahn LG et al. *JAMA Network Open.*

**eTable 6.** Adjusted odds ratios (OR) and 95% Confidence Intervals (CI) from logistic regression models for predictors of thinking of becoming pregnant among those who were NOT thinking about it before COVID-19 excluding those living outside the New York City metro area (n=817).		
	Thinking of becoming pregnant vs. still not	
	**Adjusted OR (95% CI)**	**p**
Maternal age	0.95 (0.89, 1.02)	0.15
Hispanic (vs. not Hispanic)	0.32 (0.12, 0.86)	0.02
< College degree (vs. ≥ college)	1.51 (0.53, 4.30)	0.44
Number of children in the home	0.67 (0.42, 1.06)	0.09
COVID diagnosis (self) (vs. not)	2.27 (1.03, 5.01)	0.04
PSS-4 score	0.96 (0.84, 1.09)	0.48
Current financial insecurity	0.76 (0.50, 1.16)	0.21
Took questionnaire before June 1, 2020	0.51 (0.24, 1.07)	0.07

© 2021 Kahn LG et al. *JAMA Network Open.*
